# Active Wireless System for Structural Health Monitoring Applications

**DOI:** 10.3390/s17122880

**Published:** 2017-12-11

**Authors:** Ricardo Perera, Alberto Pérez, Marta García-Diéguez, José Luis Zapico-Valle

**Affiliations:** 1 Department of Mechanical Engineering, Technical University of Madrid, 28006 Madrid, Spain; alberto.perez.calleja@etsii.upm.es; 2 Department of Construction and Manufacturing Engineering, University of Oviedo, Campus de Gijón, 33203 Gijón, Spain; garciadmarta@uniovi.es (M.G.-D.); jzapico@uniovi.es (J.L.Z.-V.)

**Keywords:** wireless smart sensor network, PZT sensors, electromechanical impedance method, Structural Health Monitoring

## Abstract

The use of wireless sensors in Structural Health Monitoring (SHM) has increased significantly in the last years. Piezoelectric-based lead zirconium titanate (PZT) sensors have been on the rise in SHM due to their superior sensing abilities. They are applicable in different technologies such as electromechanical impedance (EMI)-based SHM. This work develops a flexible wireless smart sensor (WSS) framework based on the EMI method using active sensors for full-scale and autonomous SHM. In contrast to passive sensors, the self-sensing properties of the PZTs allow interrogating with or exciting a structure when desired. The system integrates the necessary software and hardware within a service-oriented architecture approach able to provide in a modular way the services suitable to satisfy the key requirements of a WSS. The framework developed in this work has been validated on different experimental applications. Initially, the reliability of the EMI method when carried out with the proposed wireless sensor system is evaluated by comparison with the wireless counterpart. Afterwards, the performance of the system is evaluated in terms of software stability and reliability of functioning.

## 1. Introduction

Interest in Structural Health Monitoring (SHM) has increased a lot in recent years as related to both research and implementation [1,2,3,4]. This is largely in response to the fact that over time all structures deteriorate and it is essential that the owner/operator has a good idea as to the extent of deterioration and its effect of remaining service-life and capacity, and has sufficient information to make a well-informed decision regarding optimality of repair. It must not be forgotten that nowadays, considering the age of civil infrastructure, the cost of maintenance and rehabilitation of this infrastructure in advanced countries represent an enormous effort in their budget. In this sense, the efficient use of monitoring technologies might be particularly suitable for controlling the condition of existing infrastructure systems with the purpose of increasing their service life and reducing maintenance costs, the results of which are essential for the development of a sustainable civil infrastructure preservation system.

Many different SHM techniques have been used over the years, based on either global or local monitoring methods [5]. Although all global approaches are usually successfully applied and well accepted [6,7,8,9], they are based on the lowest modes of vibration, and so are not appropriate for monitoring reduced sensing regions, which is necessary to develop an accurate and efficient early detection methodology. Many non-destructive evaluation (NDE) technologies can be classified as local. The majority of local NDE methods are passive, i.e., only record the response of the structure. However, active techniques are more powerful for structural monitoring. Transducers based on piezoelectric ceramic materials (piezoelectric-based lead zirconium titanate (PZT) sensors in particular) are capable of working both as a sensor and as an actuator simultaneously [10] and, therefore, allow an active monitoring of the structure. Presently, many SHM techniques with regard to piezoelectric materials have been developed to assess the safety and integrity of in-situ structures, such as acoustic emission technique, wave propagation technique, and stress/strain technique. Electromechanical impedance (EMI) and its inverse Electromechanical Admittance (EMA) techniques are considered to be one of the most promising approaches of these piezoelectric SHM techniques due to its high sensitivity to local incipient damage and low-cost. Specifically, EMI and EMA methods allow monitoring changes in structural mechanical impedance through the electrical impedance spectra captured by PZT sensors [11,12,13,14].

The assessment of the health of a localized area by using a single active-sensor is easy [15]. However, for a large-scale structure, a robust monitoring system capable of accurately detecting and localizing damage would require a dense network of sensors. Conventional approaches consist in installing wires to connect local sensors and a centralized data monitoring system. These wired systems involve a high cost associated with their management, installation and maintenance and, additionally, their deployment can take a long period of time [16]. Many of these disadvantages disappear if a low cost wireless sensor nodes network is used [17]. The elimination of cabling makes easier the deployment of the system and its manipulation decreasing dramatically the cost. Wireless sensing systems can be installed and uninstalled rapidly making temporary, emergency deployments of health monitoring systems possible at relatively short time. Therefore, they constitute an attractive and economical alternative to the wired conventional systems and offer a very wide potential of improvement and development. In fact, in recent years a big advancement in the development of wireless sensor platforms has occurred with some of them becoming commercially available [18,19]. However, the majority of these platforms are based on passive sensors that only record the response of the structure. Only a few portable and wireless EMI monitoring systems using PZT have been developed [20,21,22]. Wireless sensors with actuation capabilities should be more powerful for monitoring structures for damage since would allow exciting the structure for its interrogation about its health when requested by the user.

**Figure 1:**
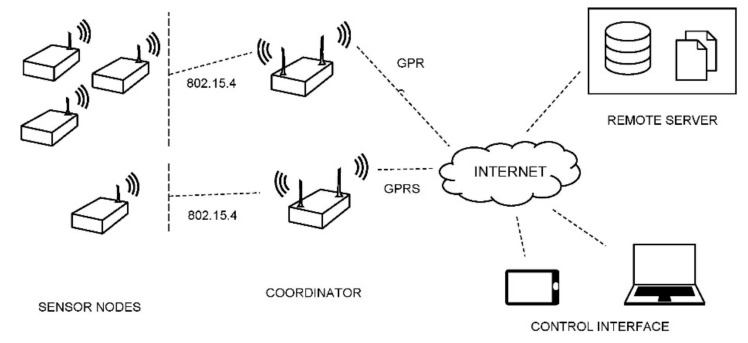
Structural Health Monitoring (SHM) wireless system.

In this paper, the design and implementation as well as the validation of a complete active wireless SHM system based on impedance measurements is presented. The system includes one or several sensor nodes, a coordinator node and the user interface for remotely controlling via Internet all the system (Figure 1). The impedance measurements, captured by the PZT patches of the sensor nodes, are transmitted through the coordinator node to an external server for storage, processing and analysis. The system is scalable since new sensor nodes can be added easily to the network keeping only one coordinator node, with the purpose of covering different areas of a complex and large structure.

In the next sections, the main characteristics of the proposed system are described in more detail and some experimental studies performed with it are presented and compared with results obtained using an active wired conventional system based on EMI method.

## 2. Description of the Active Wireless System

### 2.1. Impedance Based Sensor Node

**Figure 2:**
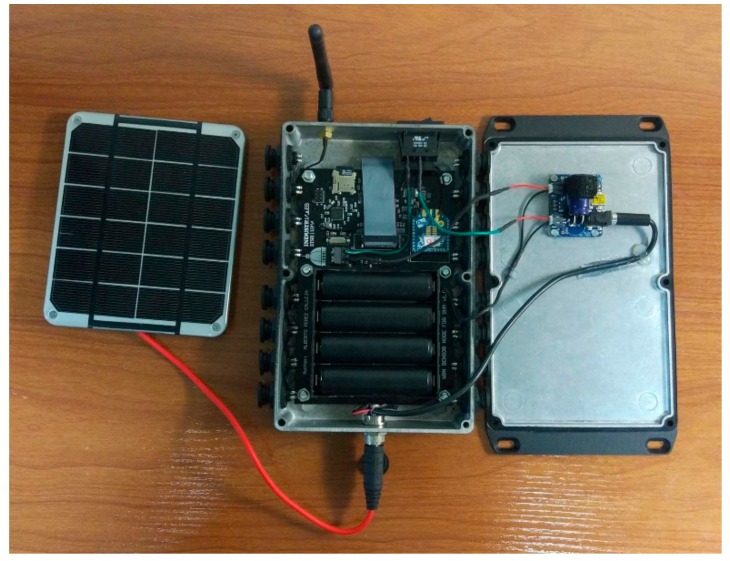
Sensor node.

The sensor node is composed of one or several PZT transducers, an AD5933 impedance measurement chip, a multiplexor, an ATmega644PA microcontroller, an Xbee S2C 802.15.4 RF module for wireless communication with the coordinator node and a realtime clock DS3221 (Figure 2). All these components are conveniently arranged on a printed circuit board (PCB).

The Atmega644PA is a high performance 8 bits microcontroller based on a Harvard architecture and specifically designed to optimize the energy consumption with respect to the velocity of the processor. Additionally, it has 64 KB of flash memory and 4 KB of static random access memory. The microcontroller controls the operation schedule, received from the coordinator node, the system and the wireless radio transmission. Additionally, together with the realtime clock DS3221 and considering that one of the most restrictive factors in the lifetime of wireless sensor network is limited energy resource of the deployed sensor nodes, it allows an efficient use of the energy by implementing a sleeping mechanism. With this mechanism an energy-conserving node hibernation protocol is set to put in sleep mode the nodes while they are not performing any operation. When a node is in sleep mode the majority of the internal hardware of the microcontroller, the impedance chip, the multiplexor and the radio module are turned off. Only the realtime clock remains turned on waking up periodically to the microcontroller to listen for a while whether there are possible messages from the coordinator node in order to determine if the node must work or sleep again accordingly. In this way, the sensor nodes work by collecting impedance information only when it is required by the user. The remaining time, they are put in sleep mode to save energy.

The core component of the sensor node directly connected to the Atmega644PA is the AD5933 impedance analyzer chip [23]. This chip includes a function generator, digital-to-analog converter (DAC), current-to-voltage converter, anti-aliasing filter, analog-to-digital converter and a discrete Fourier transform (DFT) analyzer. Furthermore, the sensor node can measure up to 16 PZT sensors through the ADG706 multiplexer connected to the AD5933. In this way, a higher density of sensors can be concentrated in specific areas of the structure without making use of an additional sensor node.

Once the AD5933 receives the order from the microcontroller to perform a frequency sweep with the appropriate parameters, the PZT sensors connected to the microcontroller are excited and the impedance values captured by them along the specified frequency range are transmitted to the microcontroller and stored in an external memory micro SD card before being sent to the coordinator node.

To perform impedance measurements under 500 Ohms with the chip AD5933, an operational amplifier with a very low output impedance in the usual range of operation has been added to the chip. In this way, PZT patches with output impedances under 500 Ohms can be used. Also, with the same purpose of obtain better measurements, a transimpedance amplifier is used instead of the one available in the chip AD5933.

Solar panels and rechargeable batteries are used as the energy power system on the sensor nodes. Batteries provide a reliable solution for WSS networks. However, for long-term deployments, energy harvesting through solar cells will extend the network operational lifetime. Lithium-Ion batteries allow installing up to 53.3 Wh with four 18,650 cells. The future improvement of the technology will allow increasing the energy power given by these batteries. A Power Management Integrated Circuit based on chip MCP73871 is implemented for battery charge management. Simultaneously, the regulator chip NCP186A has been used to stabilize the output voltage to 3.3 V before reaching the rest of the electronic system.

**Figure 3:**
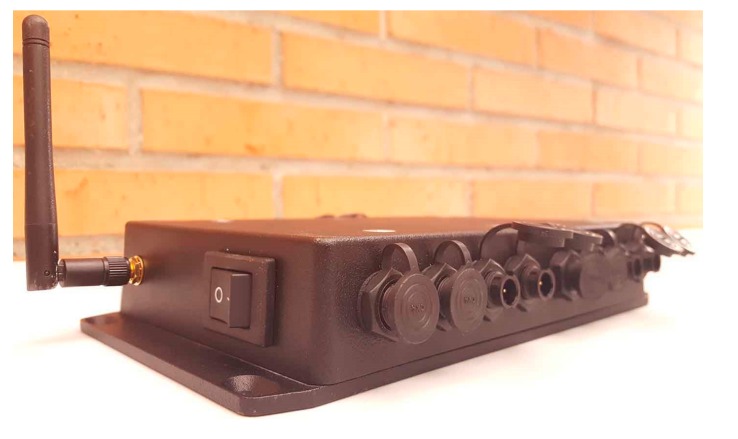
Sensor node enclosure.

In order to use the sensor node in an outdoor condition, the system is housed into an IP66 enclosure (Figure 3). This box protects the electronic components of the node against water and dust particles. The joint between the PCB and the enclosure is ensured with bolts. Additionally, sixteen electrical connectors linked to the PZT patches, an external antenna for the radio module and a power button have been externally added to the box.

### 2.2. Coordinator Node

The coordinator or gateway node is the main component of the complete wireless system since it is responsible of the coordination of all the sensor nodes of the network and, additionally, is the gateway providing communication with the remote external server. Because the coordinator node provides access to the WSS network, it is critical to the performance of the entire network. The coordinator node controls the network by sending messages to the leaf nodes, provisionally storing the transmitted data from the WSSN and transferring the data to the remote external server via internet. Only one coordinator node is needed for each sensor network although, if the number of sensor nodes is high, the network can be divided into sub-networks, each one controlled by one coordinator node.

**Figure 4:**
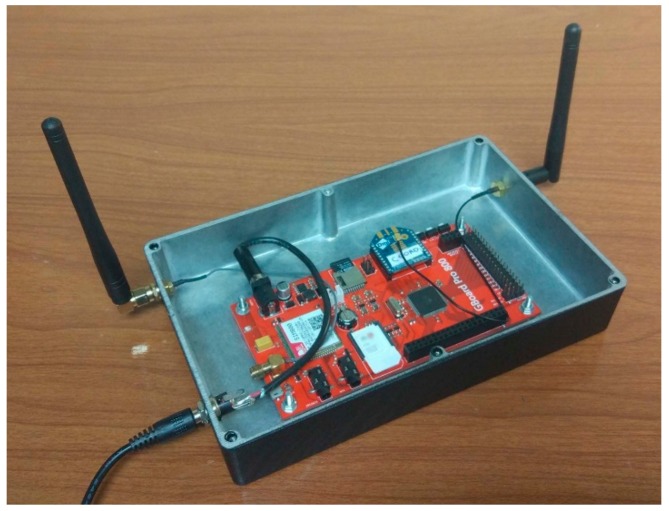
Coordinator node.

Basically, it is composed of a SIM800 Quad-band GSM/GPRS module, an Atmega2560 microcontroller and a 2.4 GHz Xbee S2C 802.15.4 radio module for the wireless bidirectional communication with the RF module of the sensor nodes. Similarly to the sensor node, the elements are arranged on a printed circuit board (Figure 4).

The GSM/GPRS module is used to enable the wireless transmission with the remote external server.

All the information related to the sensor nodes and the PZT patches to be used in one test is stored in the microcontroller memory once it has been transferred from the external server. In the same way, an external memory micro SD card is used to provisionally store the experimental data received from the sensor nodes before being transferred to the external server for their processing. 

The coordinator node is also placed in an environmentally hardened enclosure.

### 2.3. User Interface

The management of the system is performed through a middleware that acts as a bridge between a database and the sensor network. This middleware adopts a service-oriented architecture and, together with the database, is stored in an external remote server, accessed from the coordinator node with a GPRS connection. By using this architecture, the complex software system is divided into smaller and more manageable tasks. This is an attractive aspect for the application users, since, although having expertise in SHM, they usually have limited knowledge on network programming. Through a suitable user interface, the use of WSSNs of SHM applications by nonprogrammers is much easier.

By means of a Web application, the user can interact remotely with the database of the system and choose from the offered services. Some of these services are the request of execution of a new test, the management of the stored data concerning to an experimental test previously carried out, the addiction of new networks and the addition of new wireless sensor nodes to a network.

The database contains all the information concerning the wireless networks. It is achieved using five relational tables which include: (1) the name of the wireless sensor networks added and the amount of sensor nodes connected to each one, (2) all the nodes installed with their unique network address and the number of piezoelectric sensors connected to each sensor node, (3) all the tests carried out and (4) the data generated when they have ended and, finally, (5) a specific relational table to organize and coordinate the orders given through the Web application. The last one allows knowing if a request has been successfully received by the sensor network and its stage of progress.

## 3. Results

### 3.1. Damage Identification

**Figure 5:**
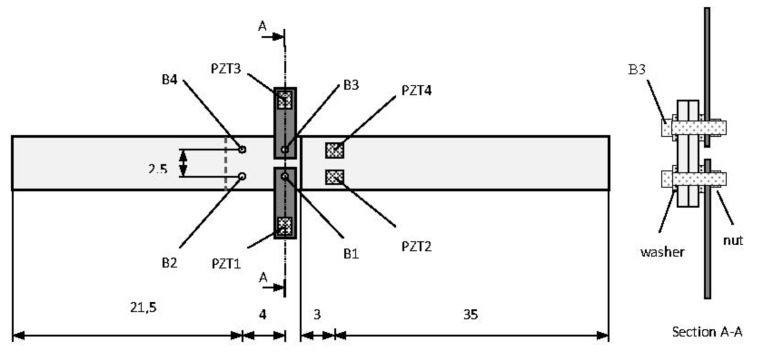
Lab-scale bolt jointed aluminium set-up (dimensions in cm).

To verify the feasibility of the proposed wireless system, a test was carried out on a lab-scale bolt jointed aluminium specimen consisting of two beams, the first one with dimensions of 45 × 5 × 0.5 cm^3^ and the second one with dimensions of 27 × 5 × 0.5 cm^3^, connected by four DIN933 M6 x 20 bolts (B1 to B4 in Figure 5). Success is based on their ability to capture data representative to identify the health of the structure being analyzed.

**Figure 6:**
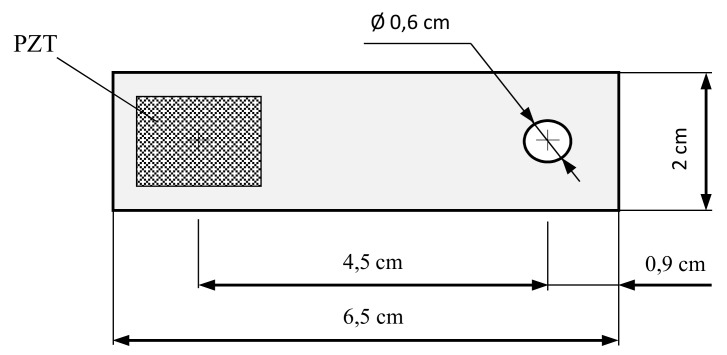
Piezoelectric-based lead zirconium titanate (PZT) interface.

Two identical P-876.SP1 DuraAct Patch Transducers [24] were bonded to one side of the lap joint (PZT2 and PZT4 in Figure 5), by using an epoxy adhesive Loctite^®^ Hysol^®^ 9455 A&B. In the same way, two PZT-interfaces made of aluminium and size of 6.5 × 2 × 0.1 cm^3^ were introduced on the bolted connections B1 and B3. These interfaces are equipped with a bonded PZT sensor (PZT1 and PZT3 in Figure 5) and their geometry is shown in Figure 6. Generally, the effective frequency range sensitive to damage changes depends on the target structure and is usually unknown for a real structure. Therefore, it is a disadvantage for the application of the impedance-based method. If a PZT-interface is installed at the joint and clamped by bolt head and connection splice, it will behave like a cantilever plate whose fixed boundary condition will depend on the contact pressure of the bolted joint [25]. Any bolt-torque reduction will cause a change in the damping and stiffness at the fixed boundary and, therefore, a change in the impedance captured by the PZT sensor. Furthermore, considering the interface behaves like a cantilever plate, these variations will occur in low frequency range.

**Figure 7:**
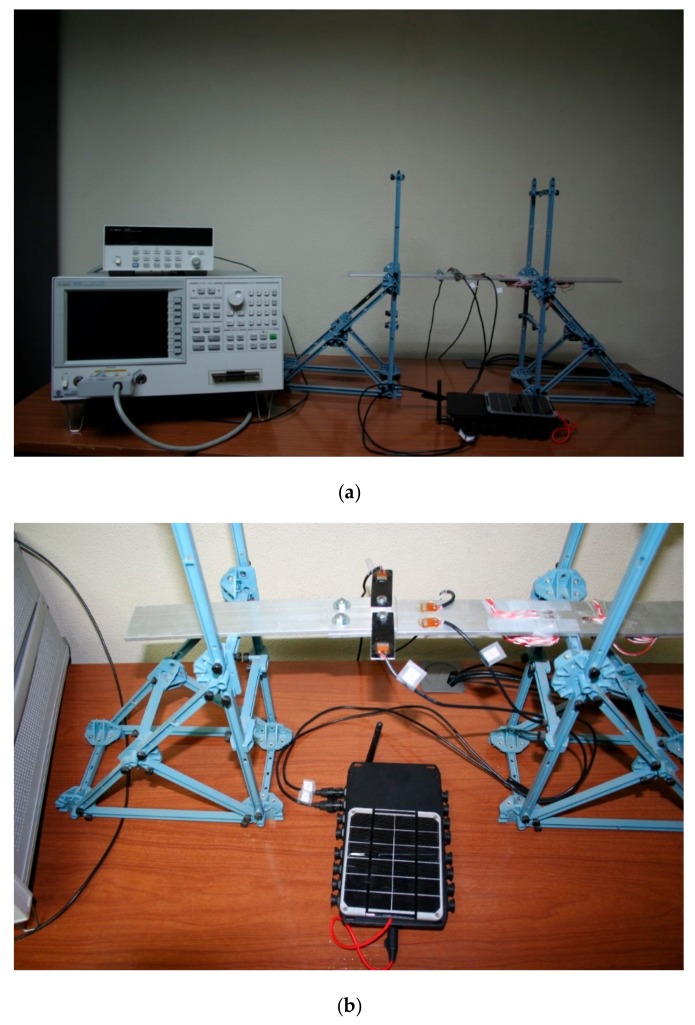
(**a**) Experimental set-up; (**b**) Detail of the joint.

To evaluate the ability of the implemented wireless system to control a structure, two sensors, PZT3 and PZT4, were connected to the commercial impedance analyzer HP 4192A from Agilent while the other two, PZT1 and PZT2, were controlled with the proposed wireless system (Figure 7). In this way, the wireless sensor node can be assessed by comparison with the commercial wired system since EMI method controlled with the wired system had been previously validated [15,26].

Three different stages to detect the damage due to loose bolts were checked: (a) All bolts were fastened to 12.5 Nm; this stage can be considered as no damage stage (S1), for which three different and independent measures were taken and used as raw data (S1-1, S1-2, S1-3); (b) State of damage (S2): Bolts 1 and 3 were loosened by 90 degrees which cause the bolts lose almost all the torque (0 Nm), this state of damage was also measured three times (S2-1, S2-2, S2-3); (c) Third state of damage (S3): All bolts were refastened again to the initial healthy condition (12.5 Nm) and also three measures of it were taken (S3-1, S3-2, S3-3). Although, in this case, the system was used to detect damage due to loose bolts in different stages, conclusions might be perfectly extended to other types of damages, such as steel yielding [14], concrete cracks [21] or FRP debonding in concrete structures [27], for instance.

It is important to remark here that all the necessary measurements were taken one after another for each subsequent damage condition. In this way, for the initial S1 condition, impedances were firstly measured. Once these measurements were collected, damage for stage S2 was induced and the new stage was measured and so on. In the same way, the three different measurements performed for each health stage were always performed for three days in a row under the same temperature conditions. Therefore, no temperature compensation was carried out.

**Figure 8:**
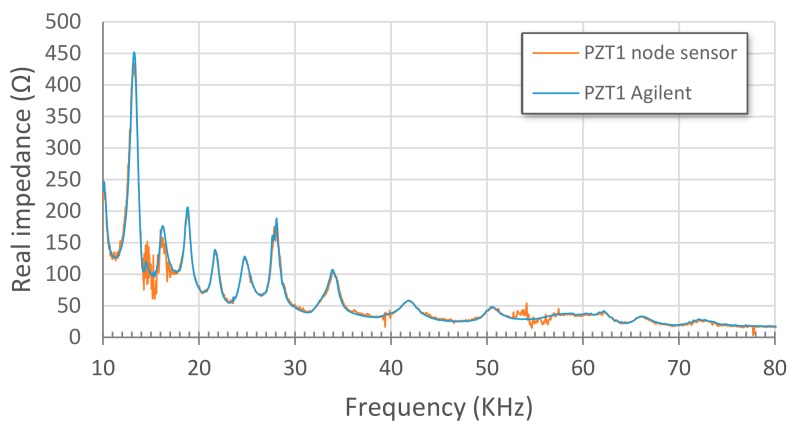
Stage S1-1. PZT1 Agilent vs. PZT1 node sensor.

**Figure 9:**
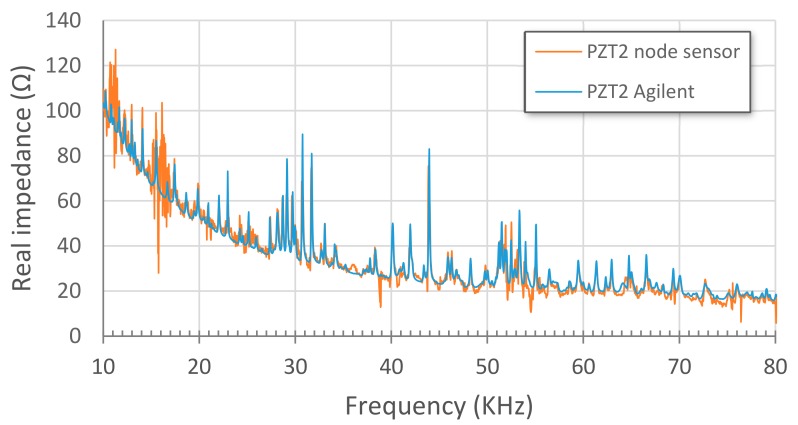
Stage S1-1. PZT2 Agilent vs. PZT2 node sensor.

Initially, EM impedance signatures captured with both a conventional impedance analyzer Agilent 4294A and the proposed wireless sensor node when connected to the same PZT patch are compared. A swept frequency range from 10 to 80 kHz was used in the tests. The comparison is made for sensors PZT1, bonded to a PZT-interface, and PZT2, directly bonded on the aluminium specimen, and for S1-1 stage (Figure 5). Figure 8 and Figure 9 show this comparison by using the real part of impedance since it gives more information and is more sensitive to damage. Although their magnitudes can differ, the characteristic peaks of the resonant frequencies of the structure show a good matching between both systems, which supports the use of the proposed wireless system for SHM applications. Furthermore, as expected, the resonance peaks are more clearly defined and bigger changes of structural impedance are obtained when the PZT-interfaces are used.

**Figure 10:**
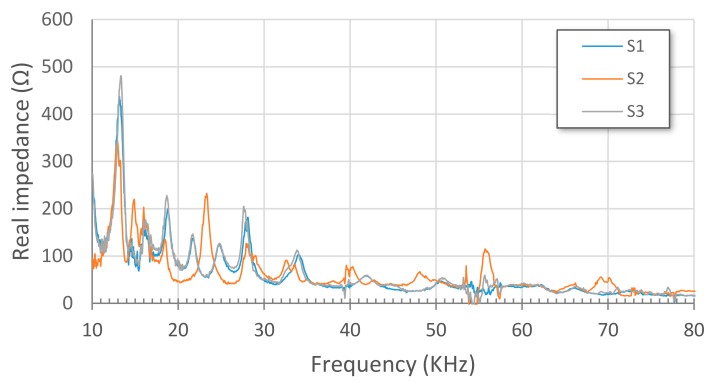
Impedance signatures from PZT1.

**Figure 11:**
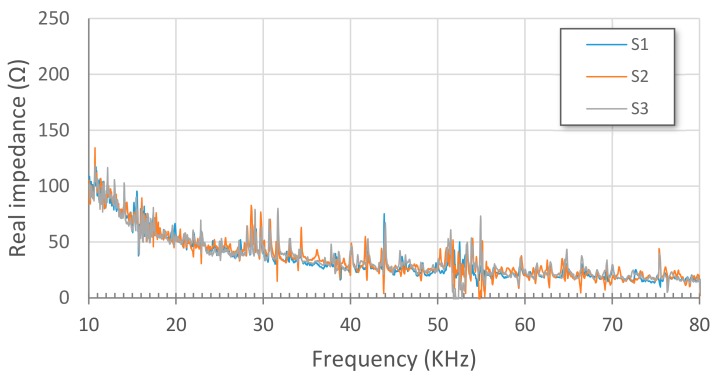
Impedance signatures from PZT2.

Figure 10 and Figure 11 show impedance signatures extracted from PZT1 and PZT2 patches, respectively, for the three considered health stages, S1 to S3. Plotted values are obtained from an average of the three measurements made for each health stage (Sx-1, Sx-2 and Sx-3). As expected, some resonance peaks shifted leftwards from S1 to S2 stages due to the loosening of some bolts. Furthermore, except for some slight variations, a good matching is reached between S1 and S3 stages since the bolts were refastened again for this last stage.

Once the EM impedance is obtained for each of the damage scenarios, an appropriate damage indicator should be used that allows a suitable analysis and quantification of the health condition of the corresponding structure. The root mean square deviation (RMSD) is the most commonly used indicator to assess damage [28], and is computed from the difference in the impedance values at each frequency point as

(1) RMSD(%)=∑i=1N(Re[Zi1]−Re[Zi0])2∑i=1N(Re[Zi0])2·100

where Re[Zi0] is the real part of the impedance of the PZT measured at a previous stage, which might agree with the healthy condition of the structure; Re[Zi1] is the corresponding value at a subsequent stage, which might agree with a post-damage stage, at the ith frequency point and N is the number of frequency points at the sweep band. For the RMSD index, the larger the difference between the baseline reading and the subsequent reading, the greater the value of the index denoting changes in structural dynamic properties which can be due to damage.

Table 1, Table 2, Table 3 and Table 4 show the RMSD values of EMI signature from the four PZT patches. The left column shows the health stage taken as baseline while the upper row shows the analyzed stage. All the possible stages have been taken as reference. The lowest values (green colour) are computed when subsequent measurements without introducing changes in the health stage of the structure are used. i.e., measurements between S1-1, S1-2 and S1-3. Changes between S1 and S2 stages (orange colour) and between S2 and S3 stages (red colour) are clearly detected. These changes are more evident than when stages S1 and S3 are compared since for these two stages the bolts are fastened. These conclusions are valid for all sensors. On the other hand, as expected, sensitivity to changes of mechanical impedance is higher in sensors on PZT-interfaces. When sensors bonded on the specimen are used, RMSD values are considerably smaller than in the previous case due to the smaller changes of structural impedance captured from PZT2 and PZT4. Additionally, the sensitivity to changes is higher for the wireless system (Table 1 and Table 2) than for the wired system (Table 3 and Table 4). Measurements captured with wireless sensors are noisier and, therefore, less repetitive. This provides higher RMSD values for these sensors even when applied to two consecutive measurements associated to the same health stage (Green cells in Table 1, Table 2, Table 3 and Table 4). However, in spite of it, predictions obtained with the wireless system are suitable and in agreement with the reality.

Finally, in order to be able to properly classify the different health scenarios into cluster groups according to the similarity or dissimilarity of their profiles, a dendrogram or hierarchical tree for each PZT is built using as distance matrix the RMSD values previously computed. By using a dendrogram, similar observations are grouped in a same cluster involving that small variations occurred between the observations belonging to the same cluster.

**Figure 12:**
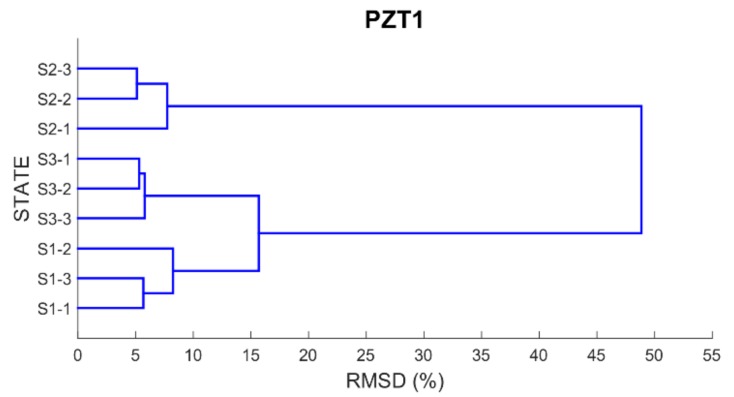
Dendrogram for PZT1.

**Figure 13:**
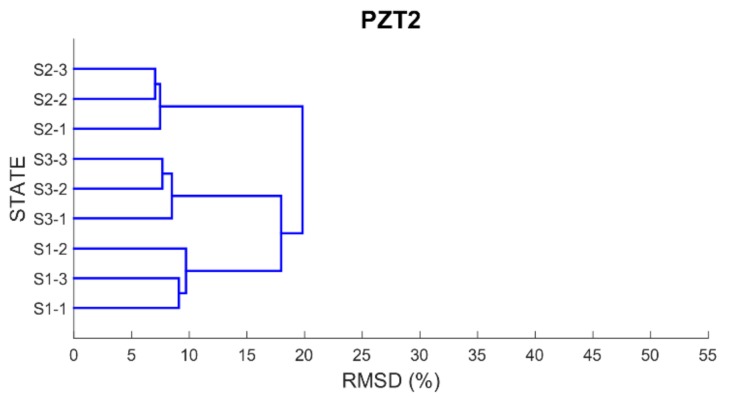
Dendrogram for PZT2.

**Figure 14:**
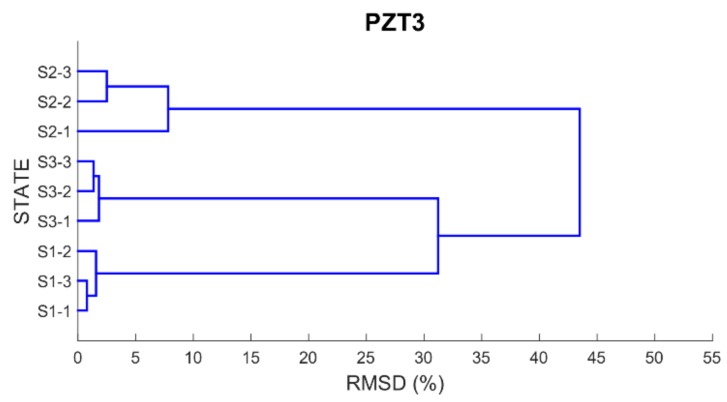
Dendrogram for PZT3.

**Figure 15:**
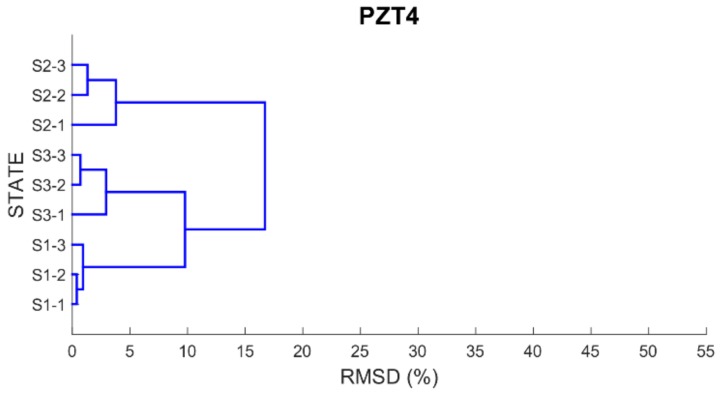
Dendrogram for PZT4.

Figure 12, Figure 13, Figure 14 and Figure 15 show these trees for the four PZT patches. In each hierarchical tree, the subdivision of the branches displays the correlation or relatedness among the different measurements. According to this, three main cluster groups (the most internal ones), coincident with the three health stages, are clearly identified for each sensor. Additionally, a new more external cluster grouping S1 and S3 cases is clearly identified which demonstrated that the similarity between S1 and S3 cases compared to S2 is perfectly detected. Other aspect to consider is the length of the branches connecting the different clusters. According to this, the length of the external branches linking the two big external cluster groups is longer for PZT1 and PZT3 sensors compared to PZT2 and PZT4 sensors. That means that variations between S1 and S2 stages and between S2 and S3 stages are more clearly detected with sensors PZT1 and PZT3 such as expected since both sensors were bonded on PZT-interfaces.

### 3.2. Performance of the Wireless System

In the previous section, the performance of the EMI method when carried out with the proposed wireless sensor system has been validated with some experiments by comparison with the data obtained using a wired monitoring system. The comparison has shown that the data quality of the proposed system is reliable to be used for SHM purposes. The following step is to evaluate the performance of the wireless system when applied to a more realistic scenario with several sensor nodes. The developed hardware as well as the software framework should be evaluated. Other aspects, such as power consumption and effective wireless range, should be also considered.

**Figure 16:**
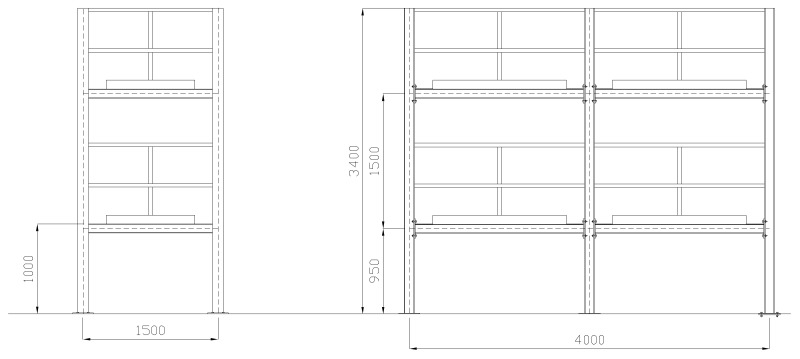
Two-storey steel frame.

For this, a middle-size two-story steel frame with two bays in the longitudinal direction and one bay in transversal one is used. The overall dimensions of the structure are 4 m length, 1.5 m width and 3.4 m height (Figure 16). All columns and beams are HEA-120 and IPN-100, respectively, of steel grade S-275. The floors of the frame are four-millimeter mm thick steel sheets connected to the beams through discontinuous welding. The foundations consist of two continuous concrete beams lying on the floor of the laboratory.

Each column consists of two pieces. They are spliced through end plates connected by four bolts 12 mm in diameter. The columns are welded to 20 mm thick plates anchored to the foundation. The beams corresponding to the transversal direction are directly connected to the web of columns by a welded-all-around fillet. In the longitudinal direction, however, the beams are connected to the flange of the columns with an eight-millimeter mm thick end plate and four bolts 10 mm in diameter.

**Figure 17:**
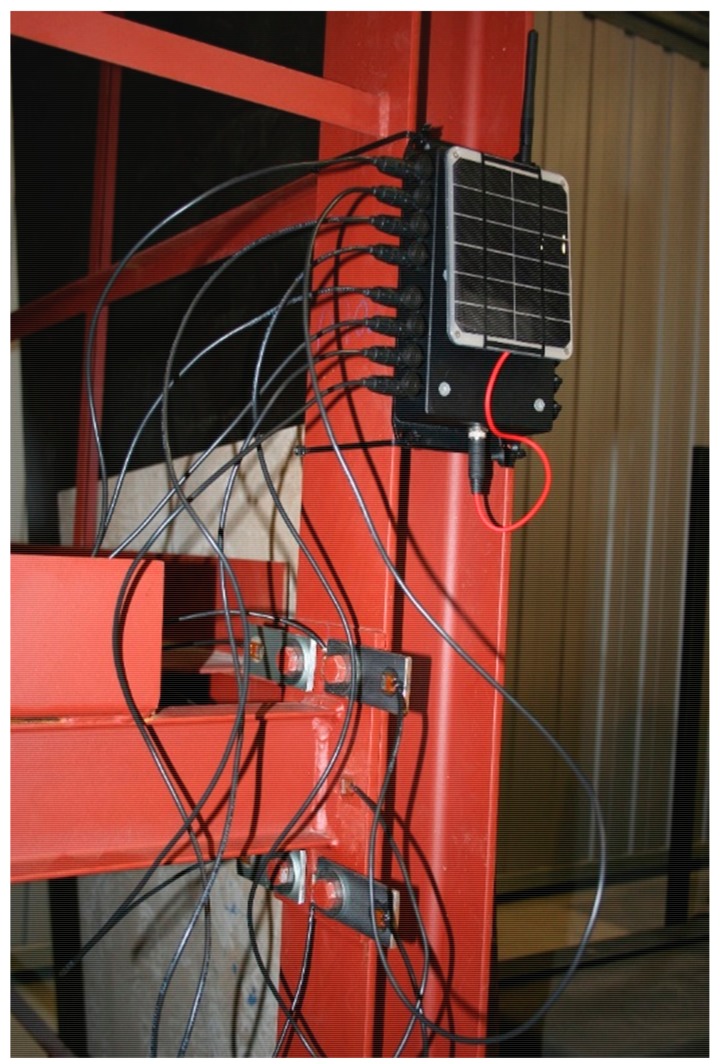
Detail of one monitored joint.

Two wireless sensor nodes were deployed on the steel frame, using clamps, to monitor two joints. At each joint, eight PZT sensors were bonded (Figure 17). This deployment is carried out to validate the suitability of the whole proposed wireless sensor network including the software developed. The configuration employed consists in one coordinator-gateway node and two sensor nodes. The gateway node and the two sensor nodes are at a remote location (several hundreds of kilometers) to the system server from which the interaction with the monitoring system is performed. Solar panels have been installed on the nodes.

**Figure 18:**
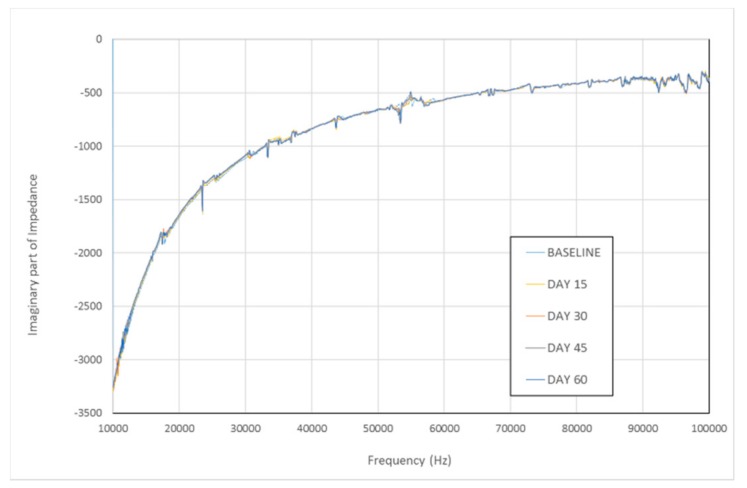
Imaginary impedance signature.

The performance of the PZT sensors, the smart sensor nodes and the coordinator node was reliable during the two months of experimental tests, enabling stable remote monitoring of the frame. Although different slight loosening of the bolts of the two joints were provided during these months, the environmental conditions of the lab were assumed to be constant during the two months of tests. To check this, Figure 18 shows the imaginary part of the impedance signatures captured by one PZT sensor each 15 days within the two months, since the real part of the impedance signatures has been reported to be more sensitive to damage than the imaginary part [29]. The temperature caused variations in the impedance signatures should be captured by their imaginary part. Figure 18 shows negligible variations among the different signatures.

Software performance was also checked. As a two-node application was being evaluated, scheduling and coordination of network tasks via software needed to be especially careful in order to avoid massive amount of data incoming to the coordinator node which might produce collapse and loss of data. Furthermore, in case of failure or malfunction of any of the nodes the system should continue operating.

A number of autonomous monitoring application tests were conducted to check the performance in the laboratory on this frame giving satisfactory results.

One of the main aspects to be considered is the communication waiting times. Long delivery times would involve a serious delay in the procedure but, furthermore, imply a significant energy consumption. This includes the sending of the measurement parameters from the remote server to the gateway and from the gateway to all the sensor nodes in the network, the data collection and, finally, the data transfer reliably back to the gateway and to the remote station for storage and subsequent processing. With this purpose, when a command was given from the remote server to initiate a test using the 16 PZT sensors installed on the two inspected joints, no collapse was observed during the data transfer to the coordinator node and the subsequent transfer to the remote station being the devoted total time of about 10 min per sensor. This operation was repeated continuously for two months and the system functioned reliably during this time enabling stable remote monitoring and communication with the coordinator node. To avoid massive transfer of data collected from the tests by the sensor nodes to the coordinator node, two sensor nodes were not allowed to send information simultaneously. Meanwhile, the data remained stored in the external memory micro SD card included on each coordinator node waiting their turn. The same procedure was followed between the coordinator node and the remote server. The storage of the data in the micro SD also allowed recovering the information from the tests if any failure occured during the data transfer.

Finally, we should comment that the two solar powered sensor nodes barely experienced decrease in the stored energy during the two months in service.

## 4. Discussion and Conclusions

In this work, the implementation of an autonomous wireless smart active sensor network has been presented. The system includes one or several sensor nodes, a coordinator node and the user interface for remotely controlling the entire system via Internet. The impedance measurements, captured by the PZT patches of the sensor nodes, are transmitted through the coordinator node to an external server for storage, processing and analysis. The system is scalable since new sensor nodes can be added easily to the network keeping only one coordinator node, with the purpose of covering different areas of a complex and large structure. Unlike the majority of wireless sensor networks implemented up to date, it is based on active wireless sensors which is more powerful for health monitoring of the structures.

The ability of the proposed wireless system to detect damage due to loosening of bolts using impedance signatures of PZT transducers has been experimentally checked. For this, values of the known statistical RMSD index have been used. The measured data and the damage identification show a good agreement with data and predictions from the existing wired system and indicate clearly the presence of damage, which verifies that the data quality of the proposed system is reliable. The system might be perfectly applied to identify other types of damage.

Performance of the system in terms of developed hardware as well as the software framework has been also verified in a several sensor nodes framework with successful results.

The wireless unit used for the SHM system can be implemented with multiple sensor nodes and costs less than 300 euros each, which is a much lower cost than traditional wired monitoring systems. Additionally, it is light weight, small size and the installation time is considerably lower than the wired counterpart.

Successful deployment of this implemented WSS network demonstrates its suitability for continuous and autonomous SHM. In a further step, the network should be checked on an exterior large-scale civil structure requiring the monitoring of different areas by using several sensor nodes.

## 5. Patents

Patent pending: P201730734 (Methodology and system of structural monitoring).

## Figures and Tables

**Table 1:** Root mean square deviation (RMSD) index (%) for PZT1.

		**Final State**								
		S1-1	S1-2	S1-3	S2-1	S2-2	S2-3	S3-1	S3-2	S3-3
**Initial State**	S1-1		7.8	5.7	46.2	46.1	46.2	16.4	15.2	14.2
	S1-2			8.6	45.6	45.4	45.6	19.1	17.9	16.5
	S1-3				46.4	46.3	46.4	16.2	14.9	14.1
	S2-1					7.4	8.1	53.4	53.1	52.1
	S2-2						5.1	53.5	53.2	52.3
	S2-3							53.7	53.4	52.4
	S3-1								5.3	6.2
	S3-2									5.3
	S3-3									

**Table 2:** RMSD index (%) for PZT2.

		**Final State**								
		S1-1	S1-2	S1-3	S2-1	S2-2	S2-3	S3-1	S3-2	S3-3
**Initial State**	S1-1		9.4	9.1	18.8	18.7	18.5	18.8	17.9	17.1
	S1-2			10.0	19.4	19.2	19.1	19.5	18.7	18.2
	S1-3				19.0	18.9	18.9	18.5	17.3	16.8
	S2-1					7.3	7.6	21.1	20.6	20.2
	S2-2						7.1	21.5	21.0	20.4
	S2-3							21.4	21.0	20.5
	S3-1								7.9	9.1
	S3-2									7.7
	S3-3									

**Table 3:** RMSD index (%) for PZT3.

		**Final State**								
		S1-1	S1-2	S1-3	S2-1	S2-2	S2-3	S3-1	S3-2	S3-3
**Initial State**	S1-1		1.2	0.8	40.3	40.4	40.3	31.3	31.6	31.2
	S1-2			1.9	39.8	39.9	39.8	31.6	31.9	31.5
	S1-3				40.6	40.8	40.7	31.1	31.4	31.1
	S2-1					7.2	8.4	46.9	47.3	46.8
	S2-2						2.5	47.0	47.5	46.9
	S2-3							46.9	47.4	46.8
	S3-1								1.8	1.8
	S3-2									1.3
	S3-3									

**Table 4:** RMSD index (%) for PZT4.

		**Final State**								
		S1-1	S1-2	S1-3	S2-1	S2-2	S2-3	S3-1	S3-2	S3-3
**Initial State**	S1-1		0.4	0.8	16.4	16.1	16.0	10.1	9.6	9.5
	S1-2			1.1	16.4	16.0	16.0	10.1	9.6	9.4
	S1-3				16.5	16.2	16.1	10.2	9.7	9.6
	S2-1					3.5	4.0	17.4	17.3	17.3
	S2-2						1.3	17.2	17.1	17.0
	S2-3							17.3	17.2	17.1
	S3-1								2.7	3.1
	S3-2									0.7
	S3-3									
